# Targeted Doxorubicin Delivery to Brain Tumors via Minicells: Proof of Principle Using Dogs with Spontaneously Occurring Tumors as a Model

**DOI:** 10.1371/journal.pone.0151832

**Published:** 2016-04-06

**Authors:** Jennifer A. MacDiarmid, Veronika Langova, Dale Bailey, Scott T. Pattison, Stacey L. Pattison, Neil Christensen, Luke R. Armstrong, Vatsala N. Brahmbhatt, Katarzyna Smolarczyk, Matthew T. Harrison, Marylia Costa, Nancy B. Mugridge, Ilya Sedliarou, Nicholas A. Grimes, Debra L. Kiss, Bruce Stillman, Christine L. Hann, Gary L. Gallia, Robert M. Graham, Himanshu Brahmbhatt

**Affiliations:** 1 Cancer Therapeutics, EnGeneIC Pty Ltd, Sydney, New South Wales, Australia; 2 Small Animal Specialist Hospital, Sydney, New South Wales, Australia; 3 Department of Nuclear Medicine, Royal North Shore Hospital, Sydney, New South Wales, Australia; 4 Cold Spring Harbor Laboratory, Cold Spring Harbor, New York, United States of America; 5 Department of Oncology, The Sidney Kimmel Comprehensive Cancer Center, Johns Hopkins University School of Medicine, Baltimore, Maryland, United States of America; 6 Department of Neurosurgery, Johns Hopkins University School of Medicine, Baltimore, Maryland, United States of America; 7 Victor Chang Cardiac Research Institute, Sydney, New South Wales, Australia; 8 University of New South Wales, Sydney, New South Wales, Australia; Bauer Research Foundation, UNITED STATES

## Abstract

**Background:**

Cytotoxic chemotherapy can be very effective for the treatment of cancer but toxicity on normal tissues often limits patient tolerance and often causes long-term adverse effects. The objective of this study was to assist in the preclinical development of using modified, non-living bacterially-derived minicells to deliver the potent chemotherapeutic doxorubicin via epidermal growth factor receptor (EGFR) targeting. Specifically, this study sought to evaluate the safety and efficacy of EGFR targeted, doxorubicin loaded minicells (designated ^EGFR^minicells_Dox_) to deliver doxorubicin to spontaneous brain tumors in 17 companion dogs; a comparative oncology model of human brain cancers.

**Methodology/Principle Findings:**

^EGFR^minicells_Dox_ were administered weekly via intravenous injection to 17 dogs with late-stage brain cancers. Biodistribution was assessed using single-photon emission computed tomography (SPECT) and magnetic resonance imaging (MRI). Anti-tumor response was determined using MRI, and blood samples were subject to toxicology (hematology, biochemistry) and inflammatory marker analysis. Targeted, doxorubicin-loaded minicells rapidly localized to the core of brain tumors. Complete resolution or marked tumor regression (>90% reduction in tumor volume) were observed in 23.53% of the cohort, with lasting anti-tumor responses characterized by remission in three dogs for more than two years. The median overall survival was 264 days (range 49 to 973). No adverse clinical, hematological or biochemical effects were observed with repeated administration of ^EGFR^minicells_Dox_ (30 to 98 doses administered in 10 of the 17 dogs).

**Conclusions/Significance:**

Targeted minicells loaded with doxorubicin were safely administered to dogs with late stage brain cancer and clinical activity was observed. These findings demonstrate the strong potential for clinical applications of targeted, doxorubicin-loaded minicells for the effective treatment of patients with brain cancer. On this basis, we have designed a Phase 1 clinical study of EGFR-targeted, doxorubicin-loaded minicells for effective treatment of human patients with recurrent glioblastoma.

## Introduction

Brain cancer treatment remains a major challenge in oncology. Despite numerous efforts, including debulking surgery, radiation therapy and chemotherapy, the prognosis remains poor [1, 2]. Moreover, surgical resection is often not a viable option due to the proximity of diffusively infiltrating tumors to vital brain structures [3]. Although the blood-brain barrier (BBB) is highly effective at restricting access of compounds to brain tissue, the invasion of tumor cells in brain tissue can disrupt its integrity [4]. Leaky vasculature is typical of tumors, resulting in an enhanced permeation-retention effect (EPR), which promotes enhanced accumulation of nanoparticles in tumor tissue compared to normal tissues [5]. For intracranial tumors, however, the EPR effect is weaker than that observed in peripheral tumors [4], which reduces extravasation of small particles from the leaky vasculature [5, 6] and limits therapeutic efficacy of systemic chemotherapy and targeted therapies in brain tumors [7–9]. Moreover, drug entry into tumors may be limited by the over-expression of drug efflux pumps by tumor cell membranes. Systemically administered drugs thus have to cross these multiple barriers prior to entry into brain tumor cells [9].

Numerous approaches have been investigated to circumvent these barriers, including direct injection into the tumor, delivery via the cerebrospinal fluid and development of direct targeting antibodies, but few have proven to be successful for the treatment of brain cancers [7, 8, 10]. Nanoparticles have been considered as potential carriers of drugs across the BBB to target tumors, including liposomes, polymeric nanoparticles, solid lipid nanoparticles, polymeric micelles and dendrimers [11]. One strategy is the use of coated nanoparticles [12, 13] that bind receptors on BBB-associated endothelial cells and then transcytose through the endothelial cells into the tumor microenvironment. An alternate strategy has been to explore the passage of particles through the tiny gaps between the endothelial cells to allow passive tumor targeting via the EPR effect, or to gain tumor access via sites in the BBB that are thought to be disrupted by tumors. It has been suggested that the upper limit of the BBB pore size in the microvasculature of malignant brain tumors is only about 12 nm [14, 15] and that drug molecules would need to be 400 Daltons or smaller [8, 10] to be able to cross the BBB.

Another difficulty in developing technologies to treat brain tumors is the lack of suitable animal models that accurately mimic the complexities of human brain tumors [16]. Although rodent brain tumor models have been used in preclinical research for over 30 years, they may not provide the most accurate model to represent features of human brain tumors [17]. Comparative oncology studies in pet dogs, that have developed cancer naturally, provide an opportunity for translational studies of new cancer therapeutics [18, 19]. Recent studies have focused on spontaneous brain tumors, including glioblastoma in dogs; the latter, like their counterparts in humans, having been found to be highly invasive with diffuse spread of tumor-derived cells in the adjacent non-neoplastic brain parenchyma [16, 20]. Like human brain tumors, these canine tumors also exhibit typical histopathological characteristics, including pseudopalisading necrosis, neovascularization and endothelial proliferation [17, 20] and inflammatory cell infiltration [20]. Overexpression of EGFR, over-expression of drug efflux pumps, and extensive invasion into normal brain and peritumoral edema [20, 21] are also observed in dog brain tumors. The estimated incidence of primary brain tumors in dogs is 20 per 100, 000/year [22] and similar to humans the prognosis for dogs with brain tumors is poor. For dogs with glioma, receieving no treatment, the median survival time ranges from 6 to 13 days [23, 24]. In one study of nine dogs receiving corticosteroid and anticonvulsant therapy only the median survival was 29 days [25]. The large size of the dog brain, compared to murine and rodent models, provides a useful model for the assessment of dose volumes. Hence, the use of pet dogs with spontaneously occurring brain tumors provides an excellent animal model that may offer better assessment of the effectiveness of novel brain tumor therapeutics [16].

Previously we have reported the use of anucleate, bacterially-derived minicells to package and target chemotherapeutic drugs to tumors to result in highly effective tumor regression in mouse xenograft models [26]. These minicells were targeted to receptors overexpressed on tumor cells via bispecific antibodies (BsAb), enabling endocytosis and intracellular drug release [26]. The anthracycline, Doxorubicin (Dox), is a potent chemotherapeutic which shows activity against many different types of cancers including glioma cell lines [27–29]. The intracellular release of Dox via minicells eliminated the toxic side effects seen with systemic administration of free drug *in vivo* [26], resulting in highly significant and potent tumor regression, using 100-fold less Dox than that required to achieve the same anti-tumor effect using Doxil [26]. Further displaying the versatility of this approach, minicells were used to effectively package siRNAs or shRNAs at therapeutically significant concentrations to combat drug resistance [30], resensitizing tumors to chemotherapeutic drugs. To date, minicells have been demonstrated as safe for systemic administration over repeated dosing in pigs and mice [26].

Using the pet dog as a translational model, we investigated the potential use of minicells as a systemically administered non-invasive method of targeting Dox to naturally occurring brain tumors. Expression of EGFR is well documented in ~60% of glioblastomas in both humans [31] and dogs [32]. Minicells were packaged with Dox and targeted to the EGFR using BsAbs directed towards EGFR. Designated ^EGFR^minicells_Dox_, this therapy was validated using *in vitro* studies prior to investigating safety and efficacy in 17 companion dogs with late-stage brain cancers.

## Results

### Dox induces potent cytotoxicity in brain cancer cells *in vitro*

**Fig 1:**
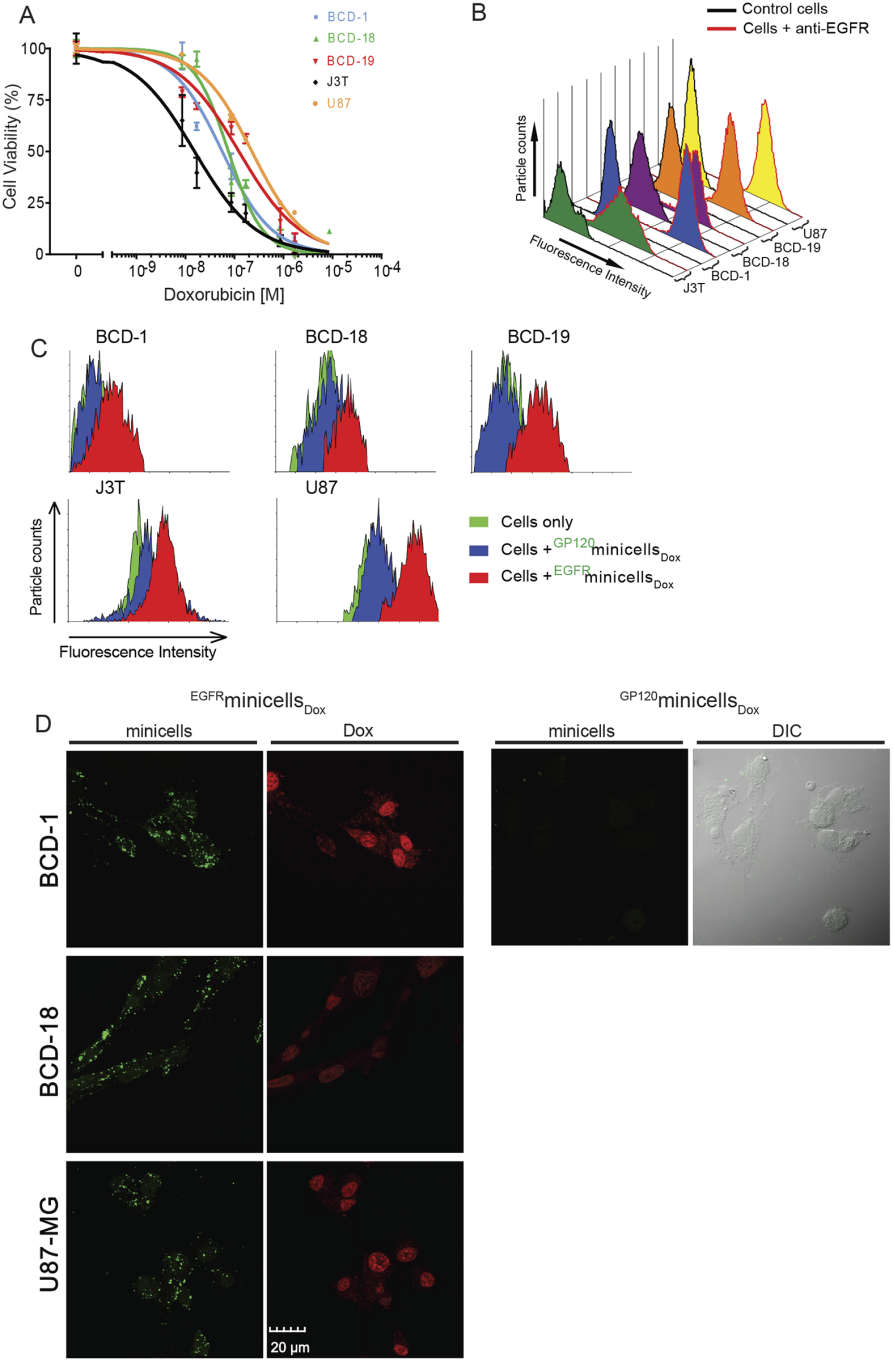
Evaluation of Dox sensitivity and EGFR expression on brain tumor cells. *In vitro* studies were performed on canine (BCD-1, BCD-18, BCD-19 and J3T) and human (U87-MG) brain cancer cells. (A) Cell proliferation (MTS) assay to determine Dox sensitivity of canine and human brain cancer cells. Error bars indicate ± SEM. (B) Quantification of EGFR on human and canine brain tumor cells (2,866,854 EGFR/cell (BCD-1); 1,465,755 EGFR/cell (U87-MG); 930,440 EGFR/cell (BCD-19); 774,352 EGFR/cell (BCD-18); and 287,622 EGFR/cell (J3T)). Control cells were treated the same except for the primary antibody. Fluorescent peaks for the control (black line) and anti-EGFR monoclonal antibody treatments (red line) are shown for each cell line. (C) Representative fluorescent peaks from fluorescence-activated cell sorting (FACS) analyses showing the efficiency of ^EGFR^minicells_Dox_ binding to canine and human brain cancer cells. In each case > 95% of the cells showed significant binding of ^EGFR^minicells_Dox_. Cells treated with non-specifically targeted ^gp120^minicells_Dox_ do not show any binding to the cells. (D) Human and canine brain tumor cells were treated with ^EGFR^minicells_Dox_ or control ^gp120^minicells_Dox_ for 3 hours. Minicells bound to the tumor cells were visualized following treatment with goat anti-mouse IgG2a-AF488 (green fluorescence) that binds to the anti-Lipopolysaccharide (LPS) component (IgG2a) of the bispecific antibody (BsAb) used to target the minicells. The right hand image of each vertical panel is visualized for Dox autofluorescence (red) and demonstrates Dox nuclear localization in most treated cells. Images were captured using a Leica fluorescence microscope. Scale bar, 20 μm.

To confirm the cytotoxic activity of Dox on brain tumor cells *in vitro*, human glioma U87 cells, canine glioma J3T cells, and tumor cell samples from dogs with brain cancer (BCD-1, -18, -19) were incubated with Dox (1.7–8,600 nM) for 72 hours and analyzed using a non-radioactive cell proliferation assay (MTS). Potent inhibition of cell viability was observed, with IC_50_ concentrations ranging from 10^−8^ to 5 x 10^−7^ M (10–500 nM; Fig 1A).

### Dog and human brain cancer cells express EGFR

The high expression of EGFR in brain cancers including glioma has led to its exploitation as a therapeutic target [33, 34], and we therefore evaluated the potential use of EGFR for targeting Dox-loaded minicells to brain tumor cells. Using flow cytometry, substantial expression of EGFR was confirmed on human brain tumor cells (U87) *in vitro* using the previously described [26] anti-human EGFR antibody (Fig 1B). Strong reactivity was observed with canine brain cancer cells including the immortalized cell line J3T and tumor biopsy samples from BCD-1, -18 and -19 (Fig 1B). Where possible, EGFR expression was also confirmed in post-mortem tumor biopsy samples from the remaining brain cancer dogs (data not shown).

### ^EGFR^minicells_Dox_ bind to dog and human brain cancer cells

Brain cancer cells were incubated with ^EGFR^minicells_Dox_ and cell binding was analyzed using FACS. After incubation with minicells for 2 hours, cell samples were fixed and processed using a fluorescently-conjugated goat anti-mouse IgG antibody. Incubation with non-specifically targeted minicells (^gp120^minicells_Dox_) caused no shift in fluorescence signal, as they were targeted to the HIV-specific protein gp120 which is not expressed in these cell populations (Fig 1C). However, a marked shift in fluorescence signal was observed in all cell populations tested after treatment with ^EGFR^minicells_Dox_, demonstrating selective binding to more than 95% of cells in each case (Fig 1C).

### Dog and human brain cancer cells efficiently take up ^EGFR^minicells_Dox_ to result in selective intracellular drug release

Binding of EGFR on brain cancer cells was visualized at a cellular level using confocal microscopy. Previously we have shown that minicells were internalized by tumor cells via endocytosis after receptor engagement, followed by intracellular drug release [26]. Here, the human U87, and canine BCD-1 and BCD-18 cells were incubated with minicells for 2 hours, and bound ^EGFR^minicells_Dox_ were visualized using a fluorescently-conjugated secondary IgG2a antibody. For both human and canine brain tumor cells, the punctate structures evident in the cytoplasm indicated internalization of the ^EGFR^minicells_Dox_ into vesicular structures (Fig 1D, left panel). Cells treated with ^EGFR^minicells_Dox_ also showed a red nuclear Dox autofluorescence (Fig 1D, left panel), indicative of intracellular Dox release and nuclear accumulation. In contrast, no binding or Dox autofluorescence was observed when cultures were incubated with minicells targeted to the irrelevant antigen gp120 (^gp120^minicells_Dox_; Fig 1D, right panel), confirming the selectivity of intracellular Dox release via EGFR-targeted minicells.

### Patient characteristics

Following confirmation of selective minicell binding and intracellular drug release *in vitro*, the safety and efficacy of systemically administered ^EGFR^minicells_Dox_ was investigated in 17 pet dogs (BCD-1 to BCD-17) with late-stage brain cancers (Table 1). Each dog received 1 x 10^10 EGFR^minicells_Dox_ (carrying ~ 8 μg Dox) via intravenous (i.v.) injection once per week, dependent upon the owner’s willingness to continue with the protocol or until the dog succumbed to the tumor or died from unrelated causes. Treated dogs were followed up for 1 month after discontinuation, or until expiry. All dogs presented with severe neurological signs and symptoms (S1 Table) consistent with those of a mass lesion involving the central nervous system, including seizures, ataxia, partial limb paralysis, partial loss of peripheral vision and aggressive behavior. Two additional dogs, BCD-18 and BCD-19 were excluded from study because they were severely ill at presentation and were euthanized.

**Table 1:** Patient Characteristics.

Patient	Breed	Sex	Age (years)	Body Weight (kg)	Diagnosis Prior to Minicell Treatment^a^	Diagnosis at Necropsy^b^	Tumor Location	Tumor Longest Diameter (mm)	Minicell Doses Received
**BCD-1**	Labrador-Retriever Cross	MN	5.62	23.18	Brain stem tumor	Choroid plexus carcinoma	Left side of posterior fossa, extending into jugular foramen	17	98
**BCD-2**	Golden Retriever	FN	7.68	33.27	Butterfly astrocytoma	No evidence of neoplastic lesions in the brain*	Corpus callosum	27	44
**BCD-3**	Terrier Crossbreed	MN	10.34	8.65	Presumed meningioma	Anaplastic astrocytoma	Arising from central skull base, extending into both sides of cavernous sinus, right middle cranial fossa and posterior fossa	20	61
**BCD-4**	Boxer	FN	10	32.9	Frontal brain tumor	Cerebral astrocytoma	Left frontal lobe (lateral cortex, sylvian fissure) and adjacent temporal lobe	30	40
**BCD-5**	Jack Russell Terrier	MN	15	6.5	Lobulated mass highly suggestive of a meningioma	Anaplastic astrocytoma	Centered on the dura overlying left frontal lobe, extending to midline	17	57
**BCD-6**	Staffordshire Bull Terrier	MN	11.24	22.66	Meningioma	Poorly differentiated malignant meningioma	Right anterior cranial fossa	30	20
**BCD-7**	Maltese Terrier	MI	9.63	4.84	Skull base meningioma	Skull base meningioma	Cavernous sinus, posterior clivus, projecting superiorly to elevate the floor of the 3^rd^ ventricle	20	20
**BCD-8**	Boxer	MI	7	23.5	Glioma	Astrocytoma	Left anterior cranial fossa	30	13
**BCD-9**	Boxer	FN	11.17	22.4	Brain stem tumor	Epithelioid meningioma	Left anterior cranial fossa	22	15
**BCD-10**	Terrier Cross	FN	10.41	9.12	Brain tumor	Intracranial granular cell tumor	Middle cranial fossa, extending over the convexity surrounding the right temporal and frontal lobes	25	17
**BCD-11**	Silky Terrier	MN	10.03	7.15	Left mesencephalic brain tumor	Mid-brain lesion. No neoplastic cells were identified in the lesion. It is possible the lesion is residual following necrosis of a neoplasm**	Left mesencephalic mass, in the left side of the mid brain at the junction with the thalamus	12	50
**BCD-12**	Bulldog	MN	4	29.7	Intra-axial pyrifom lobe mass	Oligo-dendroglioma	Left pyriform lobe	9	7
**BCD-13**	Poodle	FN	6.27	31.2	Meingioma	Psammomatous meningioma	Left frontal lobe	10	25
**BCD-14**	Border Collie	FN	8.76	23	Acoustic neuroma or a meningioma	Fibrous astrocytoma	Left cerebello-pontine angle cistern	18	7
**BCD-15**	Kelpie Cross	MN	12.02	26.8	Astrocytoma/ Glioma	No evidence of neoplastic lesion in the brain*.	Intra-axial mass left temporal lobe	10	31
**BCD-16**	Pomeranian	FN	14	5.2	Frontal brain tumor	Patient still alive.	Left frontal lobe	5	55
**BCD-17**	Cavalier King Charles Spaniel	FN	10.64	11.68	Meningioma	Posterior fossa meningioma	Left side of posterior fossa	1.8	34

FN: female neutered; MN: male neutered; MI: male intact.

^a^ Diagnosis was based on a combination of clinical signs and characteristic appearance on MRI. Tumor biopsy for histological diagnosis was deemed to be too invasive in these companion animals and was not performed.

^b^ Diagnosis determined histologically at necropsy.

* A complete response was observed in this patient, evident at necropsy with a complete resolution of the brain lesion.

** A partial response was observed in this patient, evident by the absence of neoplastic cells within a residual lesion.

Seven neutered males, eight neutered females and two intact males were enrolled in the study (Table 1). The mean weight of dogs was 18.68 kg (range, 5.20 to 35.3 kg) and their mean age was 9.64 years (range, 4.0 to 15). As is the custom in standard veterinary care in companion animals, biopsies were not taken at the time of presentation to determine the brain tumor type. Dogs were confirmed as having a brain tumor by detailed clinical staging and MRI imaging and where possible diagnosis was confirmed histologically at necropsy (Table 1). Based on the evidence available, all dogs were confirmed as having Grade IV tumors prior to enrolment in the study. Sixteen dogs underwent necropsy; BCD-16 remained alive at the time of writing this manuscript. The cohort comprised of seven astrocytomas/gliomas, five meningiomas and one each of choroid plexus carcinoma, frontal brain tumor, left mesencephalic brain tumor, oligodendroglioma and a granular cell tumor.

### Rapid accumulation of ^EGFR^minicells_Dox_ at the core of brain tumors

**Fig 2:**
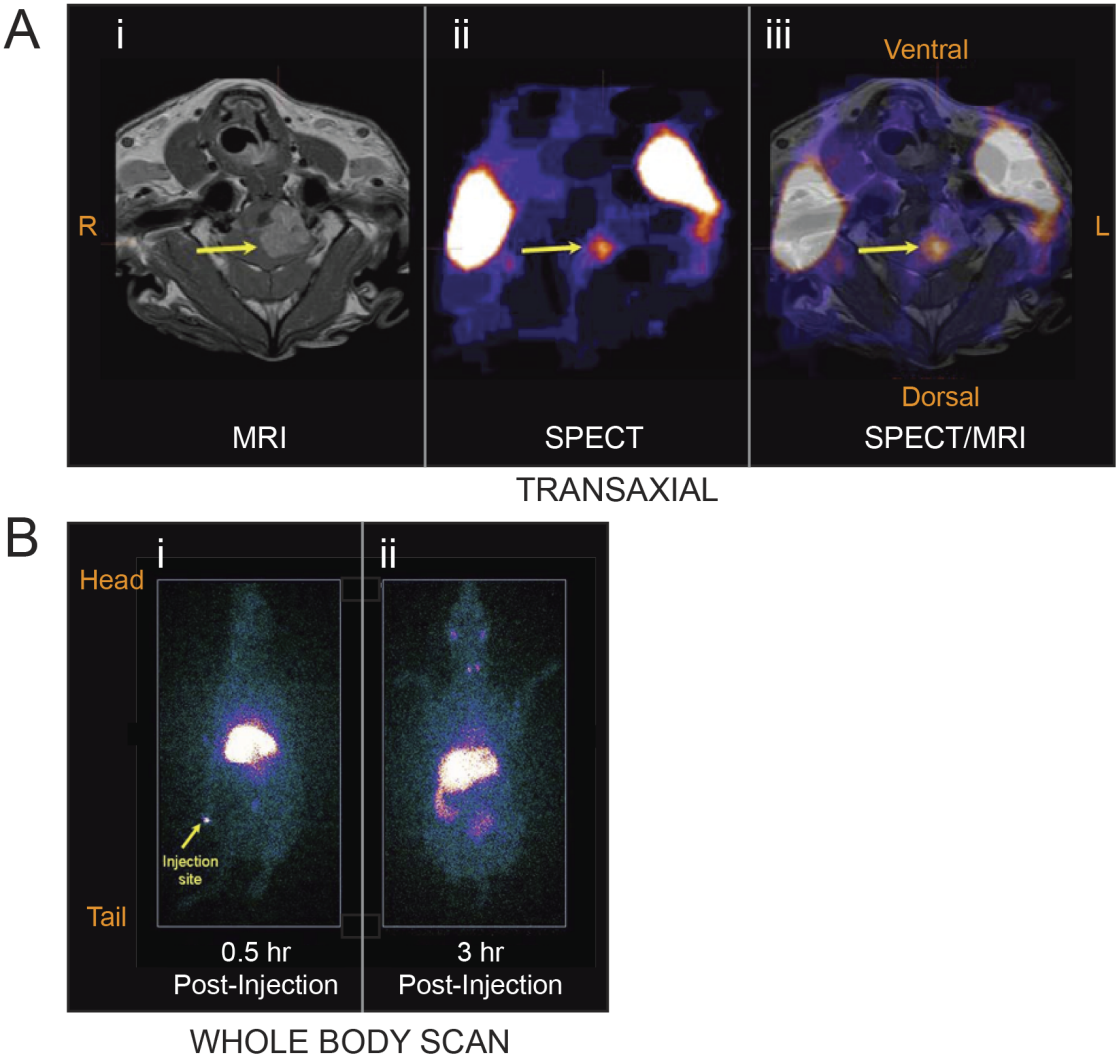
Biodistribution of systemically administered [^123^I]-labeled ^EGFR^minicells_Dox_ in patient BCD-1. (Ai) Tumor location (arrow) was confirmed by co-registered T1 post-contrast magnetic resonance imaging (MRI) in BCD-1. (Aii) At 3 hours post-minicells administration, single-photon emission computed tomography (SPECT) imaging revealed [^123^I]-labeled ^EGFR^minicells_Dox_ accumulation within the brain (arrow) in addition to a bilateral glandular uptake typically observed with iodine labels. (Aiii) A merge of MRI/SPECT images demonstrated minicell accumulation (arrow) was at the core of the brain tumor. Similar results were shown for patient BCD-3 (refer to S1 Fig). (Bi) Whole body 2D biodistribution scans in BCD-3 revealed an early uptake in the liver at 30 minutes post-administration, a common site at which bacteria are cleared from circulation. (Bii) At 3 hours post-injection, some excretion into the bowel was visible, along with uptake in the thyroid and neck glands.

To determine if systemic administration of minicells would permit access to brain tumors, biodistribution studies were performed using ^123^Iodine (^123^I) radiolabeled minicells ([^123^I]-labeled ^EGFR^minicells_Dox_). Following sedation, dogs were dosed with [^123^I]-labeled ^EGFR^minicells_Dox_ (1 x 10^10^) and imaged using SPECT and MRI, to visualize the radiolabel and tumor mass, respectively. The location of the brain tumor mass was clearly evident on post-contrast MRI images (Fig 2Ai). Importantly, cerebral SPECT images showed a focal point of [^123^I]-labeled ^EGFR^minicells_Dox_ accumulation in the brain of BCD-1 (Fig 2Aii), which directly colocalized with the core of the tumor in T1 SPECT/MRI overlay images (Fig 2Aiii). Similar findings were also observed for BCD-3 (S1 Fig).

Whole body scans revealed accumulation of [^123^I]-labeled ^EGFR^minicells_Dox_ in the liver from 30 mins to 3 hours post-injection (Fig 2Bi and 2Bii). Excretion into the bowel was visible at the later time-point (3 hours; Fig 2Bii). To a lesser extent, bilateral glandular uptake was noted in the neck, as was a small amount of thyroidal uptake (Fig 2Bii) which is commonly seen with free ^123^Iodine in radiolabeling studies.

### Systemic administration of ^EGFR^minicells_Dox_ was well tolerated in dogs with brain cancer

All dogs received at least 7 doses of ^EGFR^minicells_Dox_, 11 dogs received more than 20 doses, 4 dogs received more than 50 doses, with a maximum of 98 doses administered (Table 1). Side effects of treatment were minimal, with nausea experienced in some dogs within 2 hours of their first dose but not with subsequent treatments, and in some dogs a mild and transient rise in body temperature (0.5–1°C) was noted following administration. Despite the administration of up to 98 repeated doses, no adverse events were observed, particularly those which occur with systemic Dox infusion [35].

### Potent anti-tumor responses observed in dogs with brain cancer treated with ^EGFR^minicells_Dox_

Abnormal neurological symptoms completely resolved in all dogs after approximately 5–10 doses of ^EGFR^minicells_Dox_ (S1 and S2 Movies). Dogs were evaluated for best response on or after day 43 (dose 7). Fifteen of the 17 dogs were evaluated for response to treatment. Two of the dogs (BCD-9 and -12) could not be evaluated for responses due to owner compliance issues, however their toxicology measurements were available and used for assessment of drug safety. Overall 2 dogs had a complete response (CR) to therapy, 2 had a partial response (PR) to therapy (90% - 98.95% reduction in tumor volume), 10 had stable disease (SD), and 1 showed progressive disease (PD). The objective response rate (ORR) for treatment was 23.53% (4 of 17 dogs; 95% confidence interval, 6.8–49.8%).

**Fig 3:**
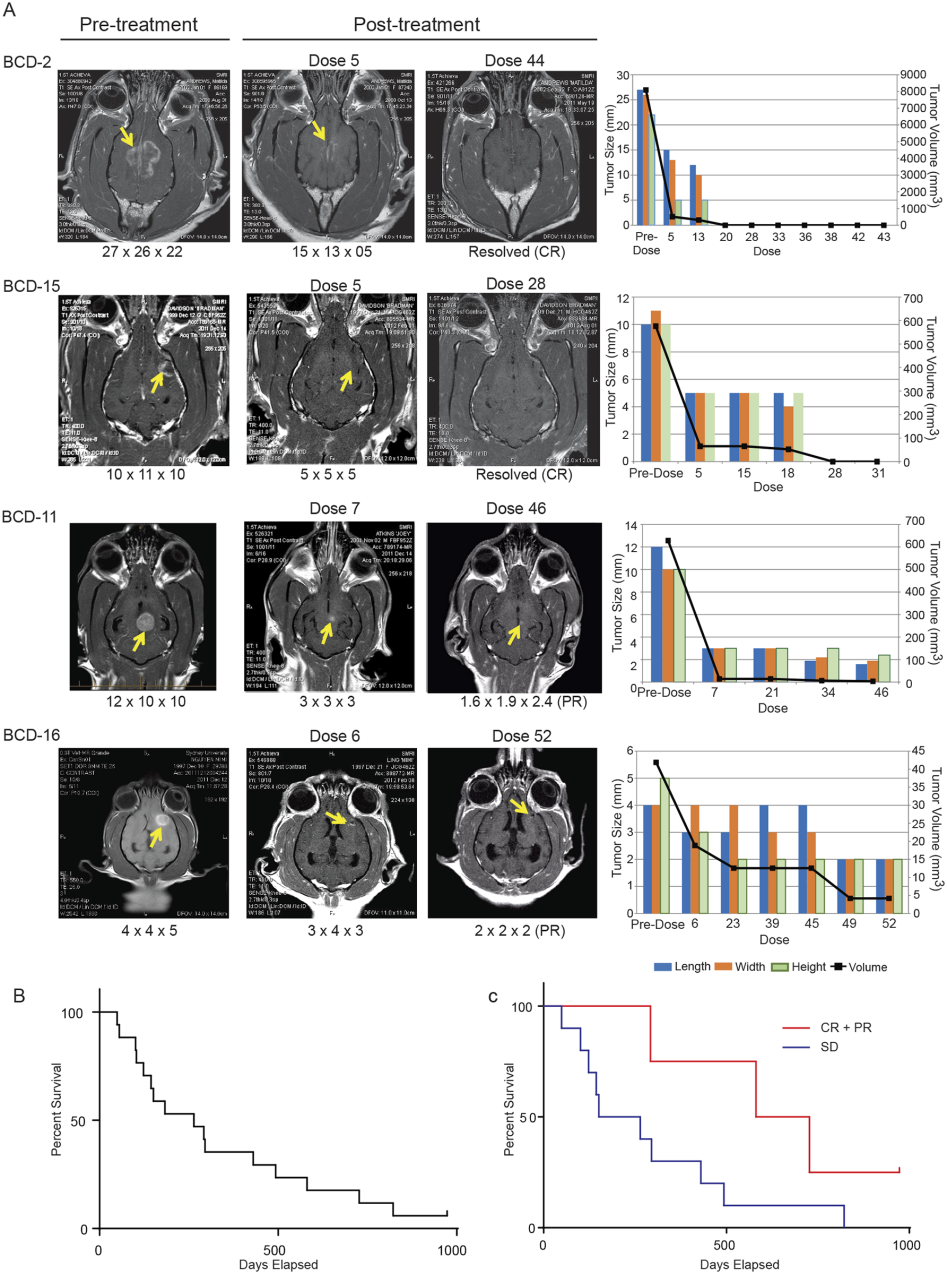
Anti-tumor response and survival of dogs with complete responses (BCD-2, -15) and partial responses (BCD-11, -16) to treatment with ^EGFR^minicells_Dox_. (A) Post-contrast axial T2 MRI sections were obtained for each dog at pre-treatment (left column), and post-treatment (middle and right column). The number of doses administered are indicated above each MRI. Tumor measurements (length, width, height) are shown in mm below each image. Measurements of tumors over time (far right), are shown for each dog, including tumor volume (mm^3^). Complete responses (CR) were observed for BCD-2 and BCD-15, and partial responses (PR) were characterized by a marked reduction in tumor size (>90% reduction in volume) for both BCD-11 and BCD-16. (B) Median overall survival for the cohort (n = 17) was 264 days (range 49 to 973). In all cases, a long-term survival response was observed. (C) Median survival for responding dogs was 654 days (CR+PR; 4/15 dogs; red line). Median survival for dogs with stable disease (SD) was 207.5 days (10/15 dogs; blue line).Post-mortem reports confirmed that death was not due to brain tumor for BCD-2, -11 and -15. BCD-16 remains alive and progression-free at the time of writing this manuscript. Red line = CR + PR survivial (n = 4). Blue line = SD survival (n = 11).

Complete responses occurred after 20 (BCD-2) and 31 doses (BCD-15), with a complete resolution of tumors evident on MRIs (Fig 3, left panel) and tumor size measurements (Fig 3, right panel). Partial responses occurred after 34 doses (BCD-11; 98.95% reduction in tumor volume) and 49 doses (BCD-16; 90% reduction in tumor volume), demonstrated by appearance on MRI images (Fig 3, left panel) and tumor measurements (Fig 3, right panel). All 4 dogs that experienced either CR or PR showed a long-term response (Fig 3) and remained progression-free.

The median overall survival was 264 days (n = 17; range 49 to 973; Fig 3B). Median survival for responding dogs (4 of 15 evaluable dogs; CR + PR) was 654 days (range, 292 to 973; Fig 3C). Of the 10 dogs with SD, the median survival time was 207.5 days (range, 49 to 822 days) including 3 dogs which survived more than 1 year. One dog (BCD-13) showed progressive disease after 5 doses, surviving to 183 days.

Upon disease progression, further treatment with ^EGFR^minicells_Dox_ did not stabilize the tumor. BCD-16 remains alive at the time of writing this manuscript, BCD-14 died a natural death during a seizure and all remaining dogs were euthanized at a humane endpoint determined by the pet’s owner in consultation with the practicing veterinarian. Post mortem analysis revealed that in BCD-2, 11 and 15, death was not due to their brain tumor.

### Liver function was maintained over repeated ^EGFR^minicells_Dox_ dosings

**Fig 4:**
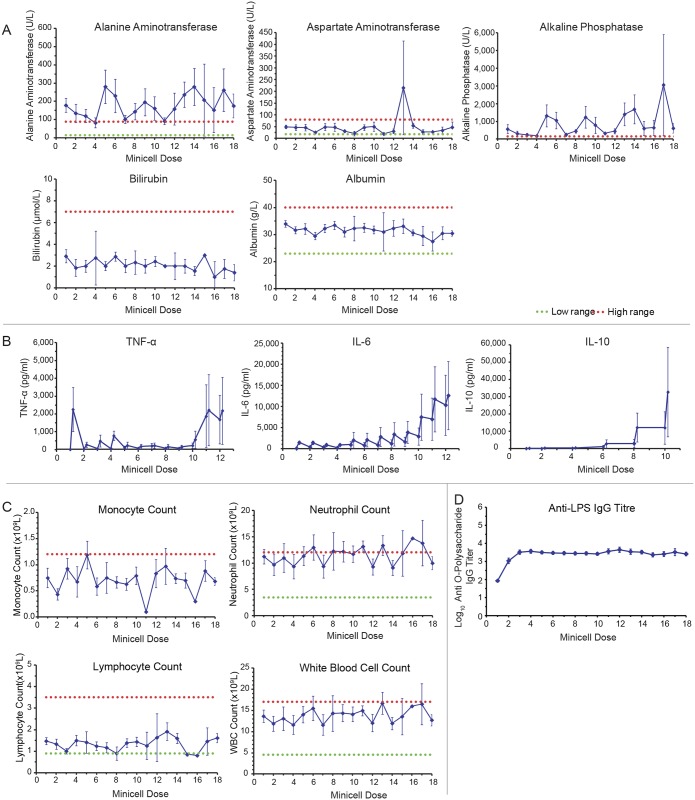
Toxicology analyses and immune responses of dogs in response to treatment with ^EGFR^minicells_Dox_. (A) Mild liver stress was indicated in all dogs by elevations in alanine aminotransferase (ALT) and alkaline phosphatase (ALP). All other liver function tests generally remained within normal reference ranges (dotted lines) over repeated doses. (B) Cytokine analysis (tumor necrosis factor alpha (TNFα), interleukin-6 (IL-6) and interleukin-10 (IL-10)) revealed sporadic elevations in some dogs, and others remained at baseline. (C) Despite repeated minicell dosing, key immune cell populations involved in responses to bacteria (monocytes, neutrophils, lymphocytes, white blood cells) remained within normal ranges. (D) A modest humoral immune response to LPS was observed to plateau after 3 doses. Data and mean ± SE are shown for up to 17 dogs at each dose of ^EGFR^minicells_Dox_. Other clinical biochemistry and hematology analyses can be found in S2 and S4 Figs, respectively.

Considering the retention of minicells at the liver, liver function tests were monitored closely. Generally, liver function tests (Fig 4A) and serum biochemistries (S2 Fig) remained similar to baseline over repeated minicell dosings. However, at the time of presentation, and prior to commencement of minicell treatment, mild liver stress was indicated in all dogs by elevations in alanine aminotransferase (ALT) and alkaline phosphatase (ALP) (Fig 4A), likely a consequence of their palliative treatment with prednisolone (0.5 to 2 mg/kg/day) and anti-seizure medication phenobarbital (2 mg/day) which are both known to induce specific elevations in ALT and ALP [36, 37]. AST levels and other liver function tests generally remained within normal ranges during the study (Fig 4A). Liver ultrasonography was routinely performed on all dogs to confirm hepatic health and rule out hepatic tumors. Food intake and body weight (S3 Fig) were maintained at similar levels throughout the study.

### Mild and transient inflammatory response observed to minicells

Considering the bacterial nature of minicells, the inflammatory response to ^EGFR^minicells_Dox_ treatment was monitored through analysis of pro-inflammatory (TNFα, IL-6) and anti-inflammatory (IL-10) cytokines. Inflammatory cytokine responses varied with each dog and showed no consistent pattern (Fig 4B). Interestingly, an elevation in the anti-inflammatory cytokine IL-10 was noted whenever spikes in TNFα and IL-6 (Fig 4B) occurred, which is linked with monocyte and macrophage activation in response to bacterial LPS [38]. In some dogs, body temperature increased from 38.5°C to 39°C within the first hour post-dosing and returned to normal by 4 hours, however no adverse clinical signs were observed.

### Immune cell populations unaffected by repeated minicell administration

Phagocytes are a key component of the immune system to recognize bacterial components in the blood [39, 40]. Previously we have shown minicells to be phagocytosed by macrophages, followed by minicell breakdown and subsequent intracellular drug release [26]. However, despite likely uptake of excess minicells by macrophages and potentially other phagocytes of the immune system, including neutrophils, hematological analysis revealed that the numbers of monocytes and neutrophils in circulation were generally maintained within normal ranges. Other hematological parameters (S4 Fig), including lymphocytes and total white blood cells (Fig 4C) were also maintained within normal ranges over repeated dosings of minicells for the duration of the study.

### Antibody responses to LPS were maintained over repeated ^EGFR^minicells_Dox_ doses

To measure humoral immune recognition to minicells in dogs, antibody titers to the O-polysaccharide component of LPS were analyzed in serum samples. In all dogs, titers of O-polysaccharide serum antibodies (Fig 4D) showed a modest increase in IgG after 3 doses of ^EGFR^minicells_Dox_ over the first 3 weeks, which then reached a plateau with no further elevation throughout the remainder of the study.

## Discussion

Many drug candidates for the treatment of brain tumors fail clinical development [8] as they are unable to accumulate selectively within the tumor tissue at levels sufficient to be therapeutically effective [41]. Here, we have reported that minicell encapsulation of Dox and targeting to EGFR permitted accumulation in canine brain tumors and demonstrated therapeutic efficacy. Most surprisingly, these marked anti-tumor responses and symptomatic remissions were observed in dogs with late-stage brain cancer.

Penetration and rapid accumulation in brain tumors, as evident in SPECT/MRI imaging studies, demonstrated minicells were able to move out of circulation and into the brain tumor microenvironment. We have previously shown that minicell accumulation at the site of tumors corresponded with release of Dox in mouse xenograft models [26]. The anti-tumor responses observed in this dog study demonstrated that minicell accumulation in the brain had occurred at concentrations sufficient to exert therapeutic benefit. The EPR effect of tumor-associated leaky vasculature has been well-established, and can permit sufficient extravasation of biocompatible macromolecules such as liposomal drug carriers at the site of tumors [42, 43]. It is likely that minicell accumulation at brain tumors observed in this study may have also occurred via the EPR effect at the tumor site.

Although studies of other bacterially-based anti-cancer therapeutics have detailed toxic side effects and adverse events [44], here we build on our previous safety studies [26] to show anucleate minicells are safe and well-tolerated in 17 dogs for up to 98 repeated doses. In contrast, the bacterially-based cancer therapeutic described by Roberts *et al* [44], consisting of Clostridium novyi-NT spores delivered via intratumoral injection to dogs with spontaneous solid tumors, produced intense reactions *in vivo* including severe inflammation, tissue abscesses, necrosis, and numerous adverse events in dogs including one case of amputation [44].

The lack of adverse events and side effects of minicells, likely due to selective intracellular release of Dox as shown here and in previous studies [26], provides a clear safety advantage. Previously used as a therapeutic target in glioblastoma, here EGFR was used as a gateway to mediate intracellular delivery of Dox, restricting systemic exposure of normal tissues to the drug. Free Dox can cause cardiotoxicity, indirect brain toxicity, or liver and kidney damage [35]. Here, encapsulating Dox within targeted minicells may help to prevent the toxic side effects of systemic Dox administration. Gaillard et al., [45] recently reported a study using glutathione pegylated liposomal doxorubicin in brain tumor-bearing mice, where moderate to severe skin reactions and weight loss was observed in the treatment group. Skin reactions, weight loss and anemia were not observed in any animal during this dog study, and these results indicate the safety and very high tolerability of minicell-derived chemotherapeutics.

Although most of these dogs did not have GBM, the most aggressive brain tumor, all dogs had Grade IV disease at the time of clinical staging. The current standard of care for brain tumors in dogs is radiotherapy, with or without surgical resection. At the time of presentation all dogs in this study had severe signs and symptoms of a mass lesion in the brain, such as seizures, partial paralysis and behavioral changes that are generally not reversible with currently available therapies. As a result, pet dogs presenting with such late-stage tumors are often euthanized rather than treated. In this study, it was not possible to have a control group since it is unethical to enroll a person’s pet animal in a study and not provide it with the study treatment. Hence, while it is not possible to conclude that the minicells provided a clear survival advantage, the stabilization/regression of the brain tumor (as seen in MRI scans) and the reversal of adverse signs and symptoms, suggest a clear clinical benefit.

Corticosteroids are widely used in the treatment of cancers, providing benefit to brain cancer patients mainly through their anti-edema effect [46]. It has been suggested that corticosteroids produce their anti-edema effect by reducing the permeability of tumor capillaries [47], resulting in a reduction of intracranial pressure and an improvement in accompanying symptoms. The dogs in this study received steroid treatment with each ^EGFR^minicell_Dox_ dose. Therefore, one could argue that the anti-tumor response observed in this study is attributed to steroid treatment and not from ^EGFR^minicell_Dox_ treatment. However, all dogs in this study had received steroid treatment prior to entering the study and had shown no clinical improvement or reduction in tumor size. During the study the dogs continued to receive the same steroid at the same rate that they had previously received, suggesting that the anti-tumor response observed in this study could be attributable to ^EGFR^minicell_Dox_.

Considering the liver is a common site at which bacteria are cleared from the circulation by Kupffer cells [40], accumulation of minicells at the liver was not surprising. Previously we have confirmed uptake of minicells by macrophages *in vitro* [26], and any potential clearance of minicells from circulation at the liver was not accompanied by liver toxicity in the current study. Furthermore, white blood cell and monocyte numbers were maintained throughout the study.

Although the response evaluation criteria in solid tumors (RECIST) can reflect rapid, high-level tumor regression in animals and humans, its ability to recognize tumor stabilization and gradual regression seen with targeted therapies is limited [48]. In contrast to traditional chemotherapy which results in immune system depression [35], immunotherapeutic agents promote immune activation to contribute towards an anti-tumor response [49]. Gradual anti-tumor effects, durable stable disease and long-term responses may provide valuable indicators of therapeutic effect for immunotherapeutic agents, aspects which are taken into consideration using immune-related response criteria (irRC) [49]. As a bacterially-derived therapeutic, it is also possible that the mild activation of the immune system by minicells in the absence of severe inflammatory responses may contribute to the anti-tumor response observed in the current study. Further research is required to confirm whether this immunostimulatory effect of minicells contributes to the anti-tumor effect observed.

Altogether, this study demonstrates that delivery of cancer therapeutics via antibody targeted, cytotoxic packaged minicells to dogs with late-stage brain tumors, is safe and extremely well tolerated. Moreover, the finding that symptoms and signs can be reversed in all animals studied, plus MRI/SPECT data demonstrating accumulation of minicells in the brain tumor microenvironment, suggest the minicell-mediated drug delivery permits entry to the brain tumor to effect anti-tumor responses. This was further noted in two dogs with complete responses and two with partial responses with greater than 90% reduction in tumor volume. Further controlled studies in humans with brain tumors are thus warranted and hold promise for the effective treatment of even late-stage disease. Together with other preclinical data, results from this study have been used to inform the design of a Phase I clinical trial of ^EGFR^minicell_Dox_ in human recurrent glioblastoma multiform (GBM) patients.

## Materials and Methods

### Study design

*In vitro* studies were performed using human and canine brain cancer cell lines in addition to canine brain tumor samples. MTS proliferation assays were used to demonstrate Dox sensitivity. FACS and confocal microscopy were used to demonstrate EGFR expression and EGFR-based targeting of minicells to brain cancer cells.

A comparative study in 17 companion dogs with spontaneous brain tumors was used as a translational model towards human trials. Each dog received a minimum of 7 doses (one dose per week) of treatment. Placebo control in this study was not feasible in companion animals due to ethical reasons where pet owners would not consent to their pet suffering from seizures to receive placebo treatment. The study was designed to determine (a) if minicells could penetrate into brain tumors, (b) anti-tumor responses in dogs with spontaneous brain tumors, (c) if dogs could tolerate repeated doses of minicells, and (d) host immune and inflammatory responses to minicells over repeated doses.

### Preparation of ^EGFR^minicells_Dox_

Minicells were produced and purified from an *Salmonella enterica* serovar Typhimurium (*S*. Typhimurium) *min*CDE- strain as previously described [26]. Dox loading, EGFR targeting, lyophilization, and dose preparation have also been previously described [26].

### Cell culture and *in vitro* studies

Human GBM-astrocytoma epithelial cells U87-MG were obtained from ATCC (catalogue number HTB-14). Primary cell lines were derived from pet dogs (BCD-18 and BCD-19) who had succumbed to their brain cancer (approved by the EnGeneIC Animal Ethics Committee. Approval number: 03/2008. Protocol title: Efficacy of Targeting EDVs Loaded with Various Chemotherapeutic Drugs in Canine Brain Cancer). The canine glioblastoma cell line, J3T [50, 51] was obtained as a gift from Dr. Michael Berens (Translation Genomics Research Institute, Phoenix, AZ, USA). All canine brain tumor cell cultures were maintained at 37°C in a 5% CO_2_ atmosphere in DMEM (Life Technologies) supplemented with 10% (v/v) FCS (Lonza); 100 U/ml each of penicillin and streptomycin, 2 mM L-glutamine and 2 mM non-essential amino acids. The human GBM-astrocytoma epithelial cells U87-MG were grown in Opti-MEM^®^ media (Life Technologies) containing 5% (v/v) FCS.

### MTS cell proliferation assay

Cells were seeded in 96 well plates (5 x 10^3^ cells/well), and incubated overnight at 37°C in 5% CO_2_. Dox (Doxorubicin HCl; Teva Pharmaceuticals) was prepared in 100 μl of relevant media (1.7 nM—8,600 nM), added to cultures and incubated at 37°C for 72 hours. Cell viability was determined using a MTS cell proliferation assay, as previously described [26]. Data analysis was performed using non-linear regression and a 4-parameter curve fit using GraphPad Prism.

### EGFR quantitation in canine and human brain cancer cell lines

Cells were harvested from culture flasks using 2 mM EDTA in PBS, and aliquots (1 x 10^6^ cells/tube) were washed and incubated in blocking solution (PBS with 2% (w/v) BSA and 0.1% (w/v) sodium azide) for 10 min at 4°C. Following incubation with anti-EGFR monoclonal antibody (1 μg/μl; IgG2a; Calbiochem) at 4°C for 45 min, R-phycoerythrin conjugated goat anti-mouse IgG (Molecular Probes, Life Technologies) was added at 4°C for 45 min with gentle agitation. For controls, PBS was used instead of the primary antibody to determine autofluorescence. Subsequent to washing in blocking solution, cells were resuspended in PBS and analyzed on the FC 500 using CXP Cytometer software (Beckman Coulter). The number of EGFR was determined by analytical flow cytometry in comparison with fluorescent R-phycoerythrin microbead standards (Quantum R-PE MESF beads; Bang Laboratories Inc). The calibration curve was generated by plotting the given number of equivalent R-phycoerythrin molecules per bead versus the log of its mean fluorescence intensity. Cellular fluorescence intensity was extrapolated onto a standard fluorescence calibration curve. The values of mean fluorescence were converted into the number of antibodies bound per cell, after subtraction of the negative control.

### Fluorescence microscopy to determine binding of minicells to cancer cells

Cells (1 x 10^5^) were plated on coverslips in 12-well plates and incubated overnight at 37°C in 5% CO_2_. Minicells (10,000/cell) were added to cultures in 300 μl of serum-free media. After 2 hours at 37°C, culture medium was aspirated, cells were washed with PBS and fixed with 4% (w/v) paraformaldehyde for 10 min. After washing, cells were blocked with 2% (w/v) BSA in PBS for 10 min at 22°C, and incubated with goat anti-mouse IgG2a-AF488 (Life Technologies) for 1 hour at 22°C. After washing, cells were imaged using a Leica DMLB fluorescence microscope with an Olympus DP70 camera and DP controller/camera software.

### Patient cohort

Seventeen companion dogs (*Canis lupus familiaris*) of various breeds and sizes (Table 1) had presented to the specialist veterinary oncology center at the Small Animal Specialist Hospital (SASH) in Sydney, Australia, for treatment of neurological symptoms. All dogs had received steroid treatment at presentation of their disease and MRI analysis showed no anti-tumor response to this treatment. Study participation was offered to the dogs’ owners after they had declined euthanasia, which is the standard of care for late-stage canine brain cancers since no effective therapy exists. This study was carried out in strict accordance with the recommendations in the guidelines for the care and use of laboratory animals of the National Health and Medical Research Council, Australia. The protocol was approved by the EnGeneIC Animal Ethics Committee (Approval number: 03/2008. Protocol title: Efficacy of Targeting EDVs Loaded with Various Chemotherapeutic Drugs in Canine Brain Cancer). Signed informed consent was obtained from all owners. All dogs underwent necropsy examination at the time of death due to any cause. Two additional dogs (BCD-18 and BCD-19) were severely ill at the time of presentation and were euthanized, however their post-mortem brain biopsies yielded tumor cell samples (glioma and astrocytoma, respectively) for *in vitro* studies.

### Diagnosis and tumor staging

All brain tumor antemortem diagnoses were based on a combination of characteristic appearance on MRI and clinical signs. Tumor biopsy for histological diagnosis was deemed to be too invasive in these late-stage brain tumor cases in companion animals, therefore, where possible diagnosis was confirmed histologically at necropsy.

Methods used for tumor staging were dependent on the histologic type and anatomic site of the tumor, and the clinical status of the subject. The criteria included physical examination, complete blood count, serum biochemistry and hematology profiles, urinalysis, coagulation profile, thoracic radiographs, abdominal ultrasound and MRI.

### Inclusion and exclusion criteria

Dogs were eligible for the study provided they had adequate performance status, and hematology/serum biochemistry analyses within normal ranges. All dogs had measurable disease at study entry but there were no restrictions on stage of disease or disease burden. In all cases, disease burden had resulted in severe neurological signs and symptoms. Adjunctive drugs for management of clinical signs of the brain tumors were allowed to continue, such as anti-seizure medication, phenobarbital (2–3 mg/kg) or prednisolone (0.75–2 mg/kg once a day, orally). During each treatment dogs received chlorpheniramine (1ml/10kg), and dexamethasone (0.2 mg/kg). Other medications that had been previously prescribed for concomitant conditions unrelated to the tumor were continued without change in dosage. Alternative therapies were not permitted during the trial period. Owner compliance issues were experienced with 2 dogs (BCD-9 and BCD-12) which prevented key tumor measurements from being obtained, although blood samples were available from these dogs for use in toxicology analyses.

### Study protocol

All dogs received 1 x 10^10 EGFR^minicells_Dox_ per dose on a weekly basis (8 μg Dox per dose, with 800,000 molecules of Dox/minicell). This dose was determined from a previous study in 2 dogs with non-Hodgkin’s lymphoma that were treated weekly with i.v Dox loaded EGFR targeted minicells for 5 or 7 doses averaging ~ 3 x 10^9^ or ~ 1 x 10^10^ minicells per dose, respectively [26]. Neither dog experienced any significant adverse reactions to treatment.

Each dose (2 ml) was administered via an aseptically placed peripheral vein catheter (left cephalic; 1 ml/min). At the time of treatment, the dogs were admitted to the veterinary clinic and blood (3 + 5 ml) was collected. Serum was collected after centrifugation at 1,580 x *g* for 15 min, and stored at -80°C until analyzed. If no toxic effects were observed at 4 hours post-dose, dogs were permitted to return home with their owners.

Dogs underwent clinical assessments and blood was collected on a weekly basis. Biodistribution studies were performed using SPECT. If a dog exhibited a change in behavior, attitude, and general wellbeing indicating that the dog was suffering then with the owner’s consent, the dog was humanely euthanized by the practicing veterinarian by i.v. administration of sodium pentobarbital (200 mg/kg).

### Biodistriubution studies

The animals were injected with approximately 40 MBq of radiolabeled [^123^I]-^EGFR^minicells and imaged at varying times over the following 4 hours. All imaging was performed using a Picker 3000XP triple-detector SPECT (Single Photon Emission Computed Tomography) gamma camera fitted with low energy, all purpose parallel hole collimators. All acquisitions used a photo peak window setting of 159 keV ± 10%. The animals were sedated with 0.1 mg/kg Buprenorphine i.v. prior to imaging. One dog was imaged non-tomographically at 30 minutes and 3 hours post-injection in a supine position to study biodistribution. Multiple planar images covering head and torso were collected in 256×256 matrices for 2 minutes per bed position at both time points and joined post-acquisition to give whole body 2D scans. All tomographic (SPECT) images were acquired in 128×128 matrices, using 120 projections of 3° radial increments (360° total) for 20 seconds per projection. All data were transferred to an off-line nuclear medicine workstation (HERMES, Nuclear Diagnostic, Stockholm, Sweden) and reconstructed using an iterative reconstruction algorithm (OSEM, 8 subsets, 4 iterations). The images were reconstructed with a software zoom of 2.0 to give voxels measuring 1.78×1.78×2.56 mm (X × Y × Z). Post-reconstruction, the images were filtered with a Butterworth filter of order 10 and cut-off of 1.25 cycles pixel-1. Previously acquired MRI scans on the dogs were imported into the workstation and the anatomical (MRI) and functional (SPECT) scans were registered in the software.

### Assessment of anti-tumor responses

Response was assessed by MRI scans approximately every 8 weeks during the course of the treatment. The MRI scans were performed with a 1.5T Phillips Achieva Scanner (Dept. of Radiology, Royal Prince Alfred Hospital, Sydney). The protocol used an 8-channel head coil or 8-channel knee coil depending on the size of the dog (small dogs used the knee coil). The sequences obtained were: Sagittal T1, axial T2, coronal gradient echo, axial diffusion pre-contrast, coronal flair, axial T1 and sagittal T1 post-contrast.

The maximum dimensions (mm) of the tumor (M/L, dorsal to ventral, craniocaudal) in each dog was provided by the veterinary surgeon based on MRI. Response to treatment was classified according to RECIST (criteria v 1.1) [52] A complete response (CR) was defined as disappearance of all known gross disease; a partial response (PR) was defined as a ≥ 50% decrease in tumor size from baseline; stable disease (SD) was designated for tumors not meeting the criteria of CR, PR or progressive disease (PD), and PD was defined as ≥ 25% increase in tumor size or the appearance of new lesions [52]. Brain tumor volume was assessed using the formula: length x width x height x (π/6). Kaplan-Meier Survival Analysis was performed and graphs were created using GraphPad Prism (V 6.0). Survival was expressed as days post-initial dose.

### Assessment of toxicity

Toxicity was assessed by owner questionnaire for signs of dysfunction of the gastrointestinal tract (anorexia, diarrhea, vomiting) and constitutional signs (lethargy/fatigue/weight loss). Hematological and biochemical toxicities were determined on a weekly basis prior to each treatment. Toxicity was graded according the Veterinary co-operative oncology group—Common Terminology Criteria for Adverse Events (VCOG-CTCAE) Following Chemotherapy or Biological Antineoplastic Therapy in Dogs and Cats v1.0 [53].

### Serum analysis: biochemistry, hematology, cytokines, anti-LPS titers

Evaluations of blood for hematological indices and serum for biochemical profiles were carried out by IDEXX Laboratories (Sydney, Australia), who also provided reference ranges for canines. Production of inflammatory cytokines TNFα, IL-6 and anti-inflammatory cytokine IL-10 were measured from canine serum samples using ELISA DuoSet kits (R&D Systems), following validation of each ELISA according to the manufacturer’s instructions. Plates were developed using TMB substrate (Sigma-Aldrich), and absorbance (450 nm) was measured using the Biotek uQuant plate reader.

To determine the host immune response to minicell-surface LPS, anti-LPS antibody titers were measured in canine serum samples using an indirect antibody-capture ELISA. Ninety six-well plates were coated with LPS purified from *S*. *typhimurium* (250 ng/well; Sigma-Aldrich) in coating buffer (10 mM sodium carbonate pH 9.6) overnight at 4°C. Wells were blocked for 1 hour at 37°C (1% (w/v) BSA in PBS), followed by addition of serum samples and overnight incubation at 4°C. After washing, goat anti-canine IgG horseradish peroxidase (HRP) conjugate (RDI) was applied to wells and absorbance (450 nm) was measured. Antibody titer was defined as the reciprocal serum dilution that gave half-maximal absorbance, and KC Junior software was used to fit a 2-parameter curve to each serum sample. Samples were analyzed in duplicate.

### Isolation of tumor cells from pet dog brain tumor biopsies

Where feasible, brain tumor biopsy samples were obtained from dogs within 2 hours of death. Tissue samples were treated for 10 min with 1 mg/ml collagenase in DMEM media containing 10% (v/v) FCS, 100 U/ml each of penicillin and streptomycin. Undigested tissue was removed by filtration through a double layer of sterile gauze swab. Collagenase digestion was stopped by diluting the cells with 5 ml media and then centrifuging at 1,200 x *g* for 5 min. Cells were washed with an additional 5 ml media followed by centrifugation, resuspension, and plated in tissue culture flasks.

### Histopathology

At necropsy, all tumors were studied histopathologically to determine tumor type. Selected tumor samples obtained post-mortem were formalin-fixed and processed by routine paraffin embedding. Sections (5 μm) were cut and stained with hematoxylin and eosin. All specimens were reviewed by a board-certified pathologist and assessed according to the classification of animal tumors [54].

## Supporting Information

S1 ARRIVE Checklist (PDF)

S1 Fig Biodistribution of ^123^Iodine-labeled ^EGFR^minicells_Dox_ in brain cancer dog BCD-3. At 3 hours post-minicells administration, tumor location (yellow arrow) was confirmed by co-registered T1 post-contrast MRI (i) in BCD-3. Biodistribution of ^123^Iodine-labeled ^EGFR^minicells_Dox_ was studied using SPECT imaging, demonstrating accumulation of radiolabel within the brain (ii; yellow arrow), which directly colocalized with the core of the brain tumor (iii; yellow arrow) in the MRI/SPECT overlay image. Some bilateral glandular uptake was also observed, which is typically observed with iodine labels.
(TIF)

S2 Fig Clinical biochemistry analysis of serum samples for all seventeen brain cancer dogs during treatment with ^EGFR^minicells_Dox_. The mean values (n = up to 17; BCD-1 to BCD-17) are shown at each dose of ^EGFR^minicells_Dox_ (x-axis). The normal reference range for each parameter is shown in each graph with red (upper limit) and light green (lower limit) lines. Data are means ± SD.
(TIF)

S3 Fig Weights for all seventeen brain cancer dogs during treatment with ^EGFR^minicells_Dox_. The weights of all dogs generally remained consistent throughout the study.
(TIF)

S4 Fig Hematology analysis of serum samples for all seventeen brain cancer dogs during treatment with ^EGFR^minicells_Dox_. The mean values (n = up to 17; BCD-1 to BCD-17) are shown at each dose of ^EGFR^minicells_Dox_ (x-axis). The normal reference range for each parameter is shown in each graph with red (upper limit) and light green (lower limit) lines. Data are means ± SD.
(TIF)

S1 Movie BCD-2 at the time of clinical staging. The video shows neurological deficits in the right hind leg. The veterinary oncologist shows that the reflex in the left hind leg is normal and if the foot is bent backwards, the reflex is for the foot to return to normal position. In contrast, when the oncologist bends the right hind leg backwards, the foot shows a complete lack of reflex action and the foot remains in the backwards position.
(MOV)

S2 Movie BCD-2 after receiving five doses of ^EGFR^minicells_Dox_. The reflex action in the right hind leg returned to normal.
(MOV)

S1 Table Clinical signs of brain cancer dogs. At the time of tumor diagnosis and staging, each dog was assessed for neurological signs of disease by the practicing veterinarian. Diagnosis was based on a combination of characteristic appearance on MRI and clinical signs. Tumor biopsy for histological diagnosis was deemed to be too invasive in these brain tumor cases in companion animals but, where possible, diagnosis was confirmed histologically at necropsy.
(TIF)
