# Supplementary figures and images for: Targeted Doxorubicin Delivery to Brain Tumors via Minicells: Proof of Principle Using Dogs with Spontaneously Occurring Tumors as a Model
Source: PLoS One. 2016 Apr 6;11(4):e0151832. doi: 10.1371/journal.pone.0151832 (PMC4822833; doi:10.1371/journal.pone.0151832)

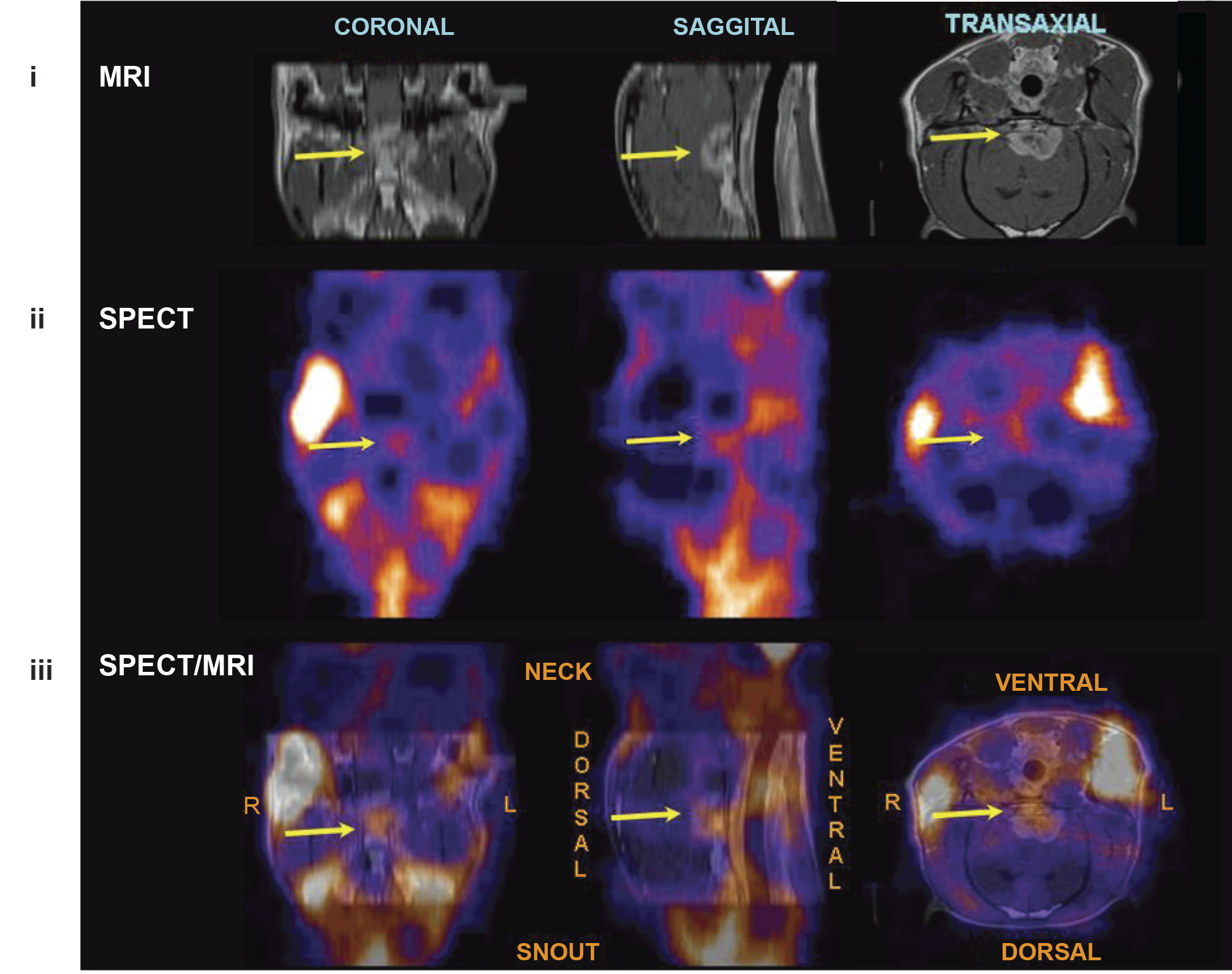

Supplement: S1 Fig — At 3 hours post-minicells administration, tumor location (yellow arrow) was confirmed by co-registered T1 post-contrast MRI (i) in BCD-3. Biodistribution of 123Iodine-labeled EGFRminicellsDox was studied using SPECT imaging, demonstrating accumulation of radiolabel within the brain (ii; yellow arrow), which directly colocalized with the core of the brain tumor (iii; yellow arrow) in the MRI/SPECT overlay image. Some bilateral glandular uptake was also observed, which is typically observed with iodine labels. (TIF) [file pone.0151832.s002.tif]

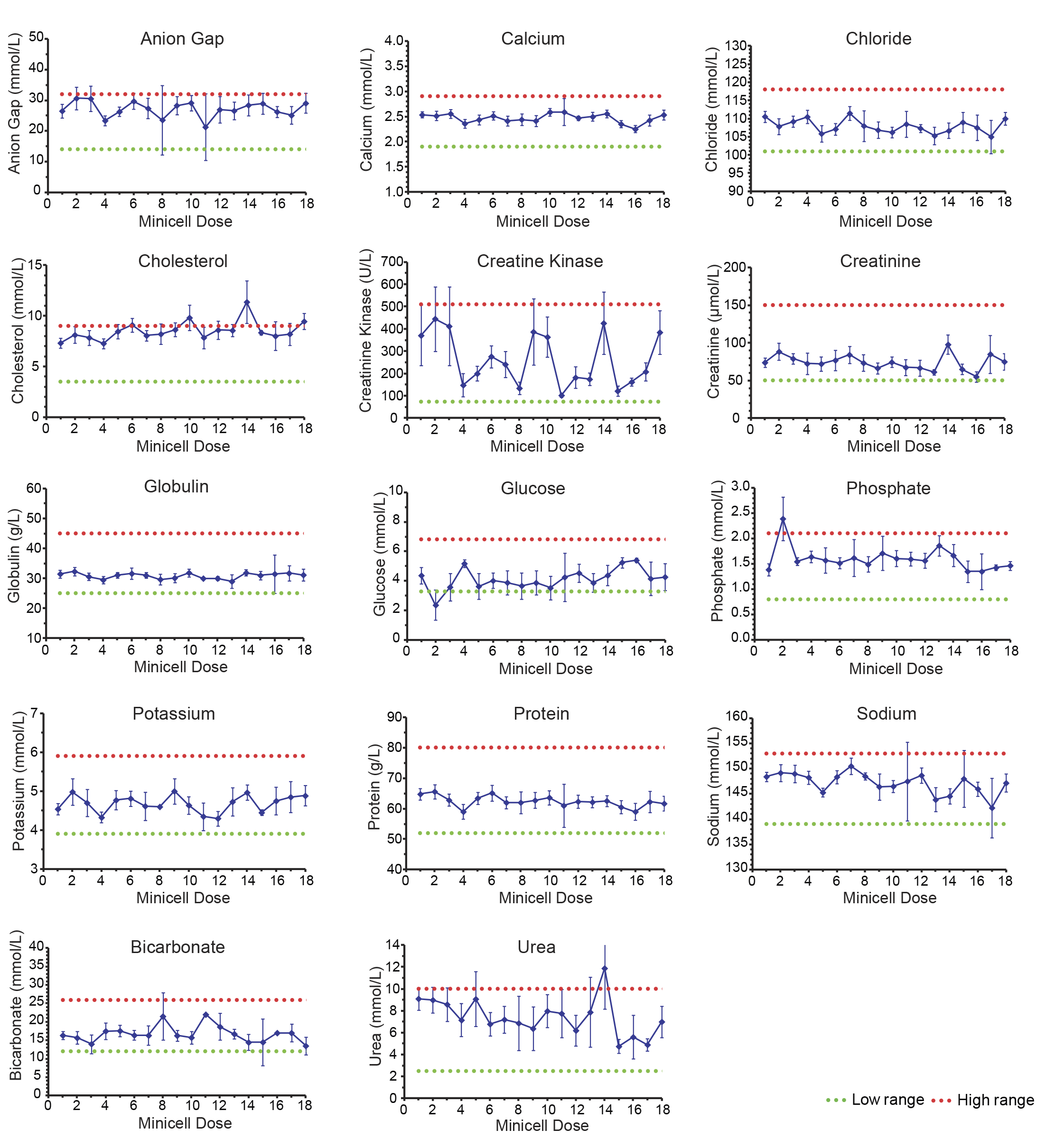

Supplement: S2 Fig — The mean values (n = up to 17; BCD-1 to BCD-17) are shown at each dose of EGFRminicellsDox (x-axis). The normal reference range for each parameter is shown in each graph with red (upper limit) and light green (lower limit) lines. Data are means ± SD. (TIF) [file pone.0151832.s003.tif]

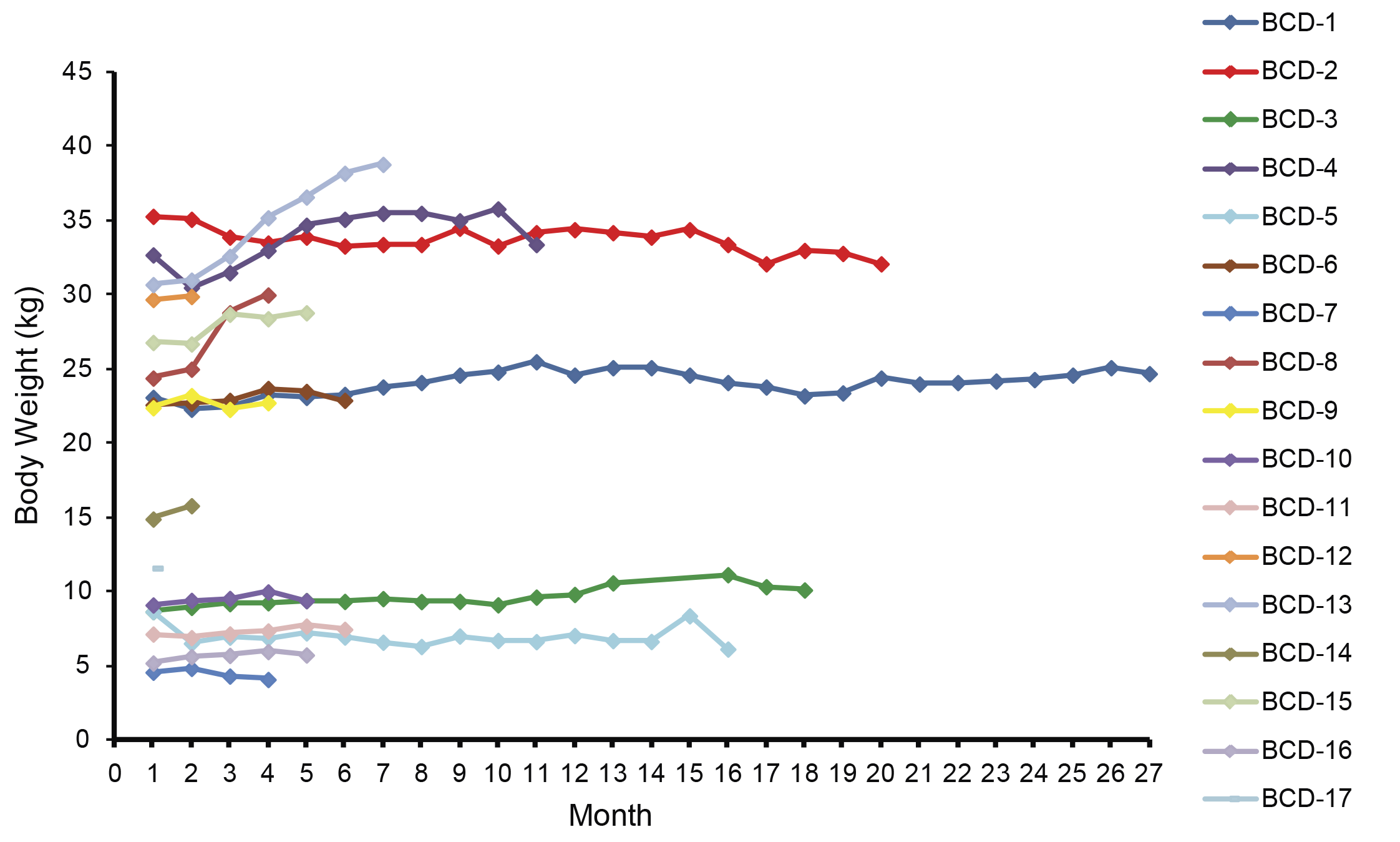

Supplement: S3 Fig — The weights of all dogs generally remained consistent throughout the study. (TIF) [file pone.0151832.s004.tif]

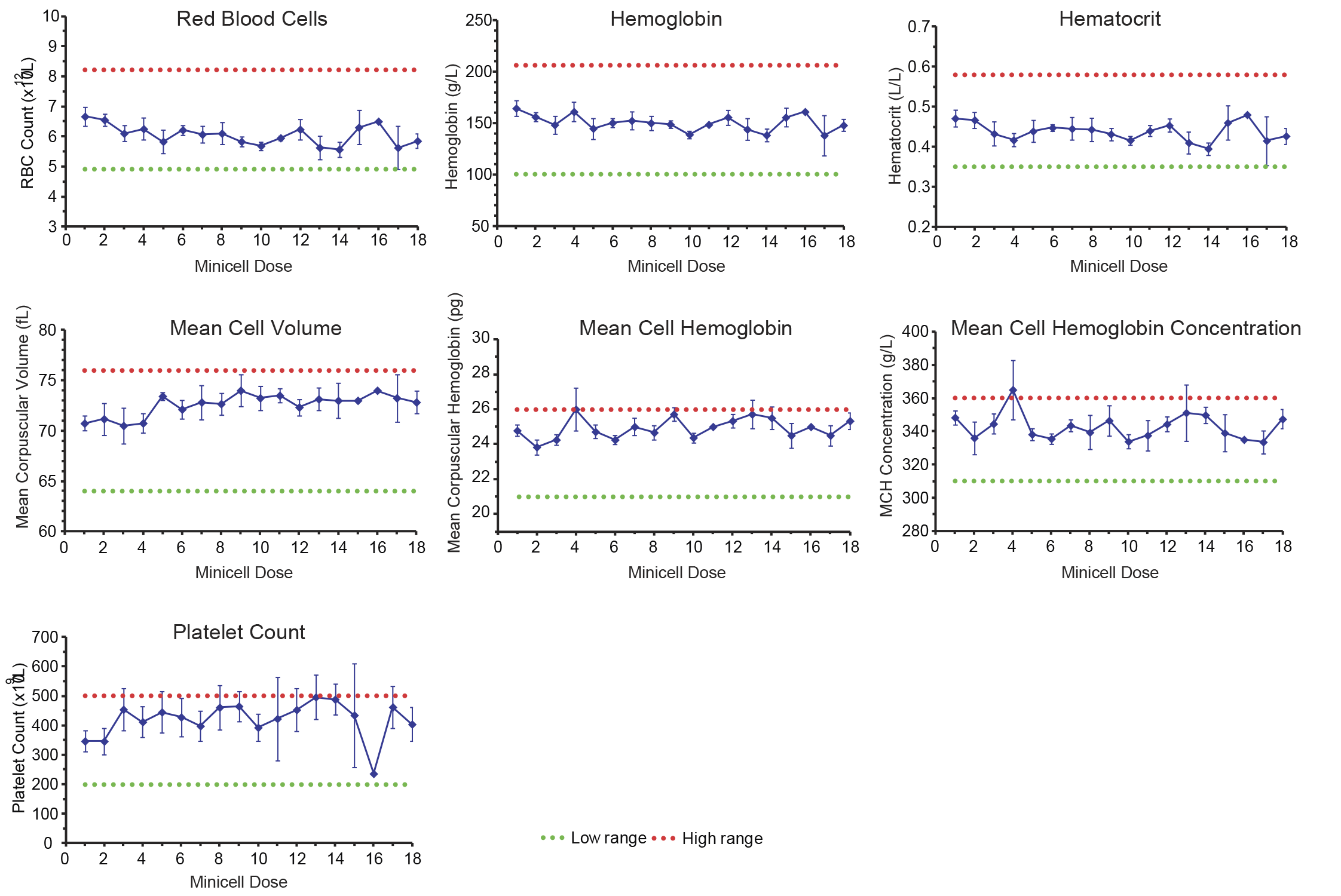

Supplement: S4 Fig — The mean values (n = up to 17; BCD-1 to BCD-17) are shown at each dose of EGFRminicellsDox (x-axis). The normal reference range for each parameter is shown in each graph with red (upper limit) and light green (lower limit) lines. Data are means ± SD. (TIF) [file pone.0151832.s005.tif]

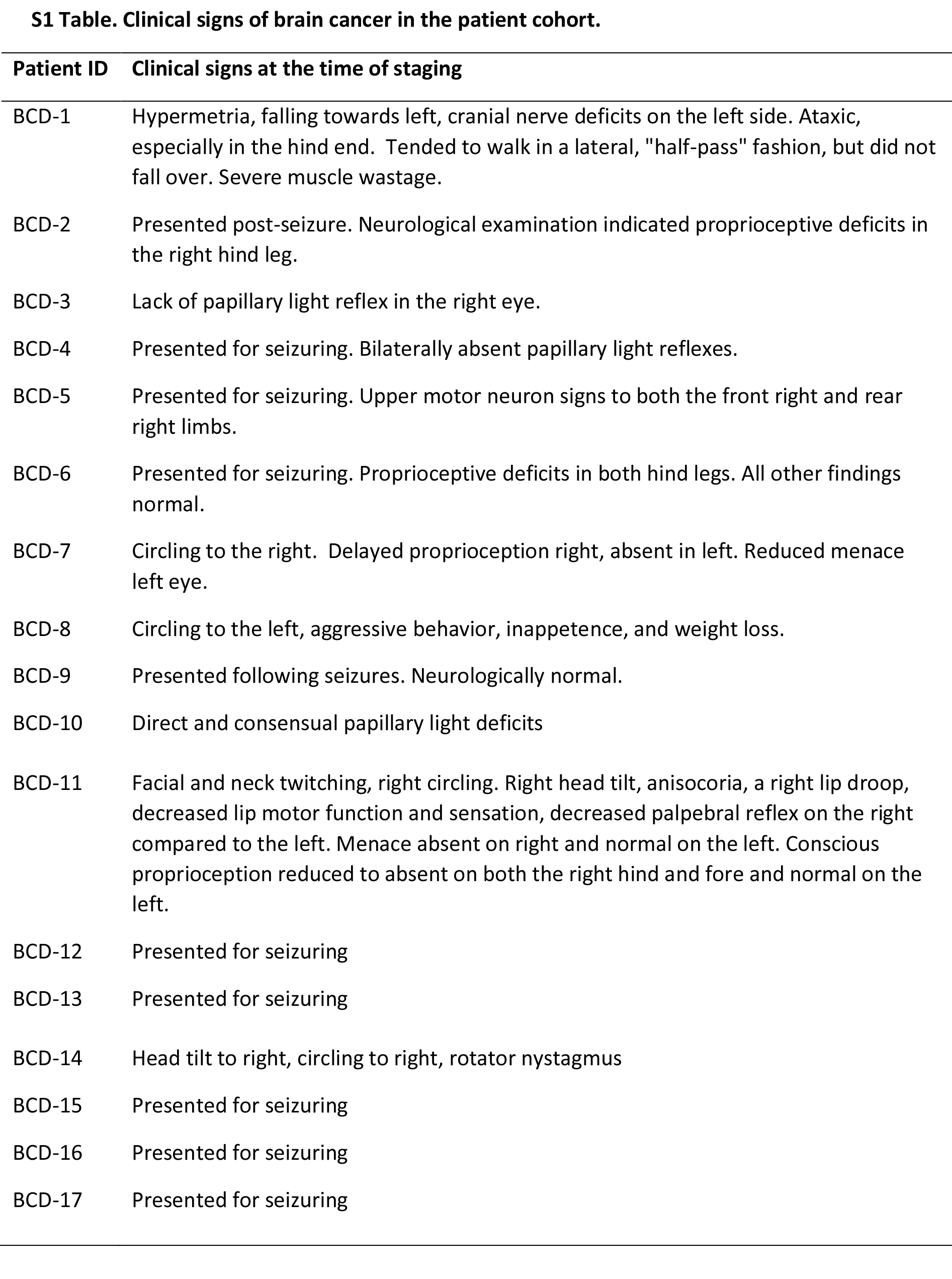

Supplement: S1 Table — At the time of tumor diagnosis and staging, each dog was assessed for neurological signs of disease by the practicing veterinarian. Diagnosis was based on a combination of characteristic appearance on MRI and clinical signs. Tumor biopsy for histological diagnosis was deemed to be too invasive in these brain tumor cases in companion animals but, where possible, diagnosis was confirmed histologically at necropsy. (TIF) [file pone.0151832.s008.tif]
